# Alobar Holoprosencephaly in a Newborn: A Case Report of Prenatal Diagnosis and a Review of the Literature

**DOI:** 10.7759/cureus.74462

**Published:** 2024-11-25

**Authors:** Kamal Chafiq, Khalil Toumi, Fatima Ezzahra Khayi, Abdellatif Daoudi

**Affiliations:** 1 Neonatology, Souss Massa University Hospital Center, Agadir, MAR

**Keywords:** alobar holoprosencephaly, craniofacial abnormalities, fetal ultrasonography, holoprosencephaly, prenatal diagnosis

## Abstract

Holoprosencephaly (HPE) is a severe and complex congenital brain malformation caused by a defect in the midline cleavage of the prosencephalon during early embryonic development. It is the most common prosencephalic malformation in humans and is categorized into three classical forms based on the severity of this cleavage defect: alobar, semilobar, and lobar HPE. A milder interhemispheric variant, called syntelencephaly, is also considered a form of HPE. This condition may arise from genetic, environmental, or teratogenic factors. Patients with HPE often present with facial dysmorphia, with severity generally correlating with the extent of brain malformation. HPE is diagnosed prenatally through ultrasound and brain magnetic resonance imaging (MRI). Through this case report and a review of the literature, we discuss the etiopathogenic and diagnostic aspects of HPE, along with the management of this congenital malformation, illustrated by the antenatal diagnosis of a newborn with alobar HPE, confirmed by brain MRI at 32 days of age.

## Introduction

Holoprosencephaly (HPE) is defined as a set of structural brain abnormalities resulting from a midline differentiation and cleavage defect in the prosencephalon during the third and fourth weeks of gestation. This condition is the most common malformation of the prosencephalon in humans [1]. It affects approximately one in 250 pregnancies; however, due to the significant number of fetal deaths, its prevalence at live birth is lower, around one in 10,000 [2].

HPE is classified into two categories based on the degree of cerebral hemisphere differentiation. The first includes the classical forms, ordered by severity: alobar, semilobar, and lobar [3]. The second category corresponds to the interhemispheric variant, also known as syntelencephaly [4]. Forebrain malformations are generally associated with facial abnormalities, ranging from severe features like anophthalmia, cyclopia, or proboscis in severe cases to median cleft lip, simple hypertelorism, or no abnormalities in milder forms of HPE [5].

HPE is a multifactorial disorder with diverse etiological causes, including environmental, genetic, teratogenic, and syndromic factors. Chromosomal abnormalities are often observed, with trisomy 13 (Patau syndrome) being the most common [6].

Children with HPE present various medical issues, including developmental delays, feeding difficulties, epilepsy, and dysautonomia. They also frequently suffer from endocrine disorders, such as diabetes insipidus, adrenal hypoplasia, hypogonadism, thyroid hypoplasia, and growth hormone deficiency, underscoring the importance of early prenatal diagnosis through fetal ultrasound for better management [1].

We report the case of a full-term female newborn from a non-consanguineous marriage and poorly monitored pregnancy, diagnosed with alobar HPE. The prenatal diagnosis was made late at 24 weeks of gestation and was confirmed by a brain MRI performed at 32 days. The guardian consented to have the patient's identity revealed in an open-access publication. A written and signed consent statement was provided to the journal.

## Case presentation

**Figure 1:**
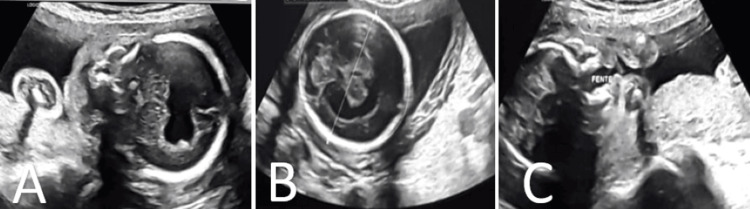
Fetal ultrasound in sagittal (A) and axial (B) views shows microcephaly, a single ventricle, a frontal parenchymal band without an interhemispheric fissure, and thalamic fusion. Sagittal view (C) shows a median cleft lip-palate

We present the case of a full-term female newborn weighing 2750 grams, born to a 24-year-old primigravida mother through spontaneous vaginal delivery. The pregnancy was poorly monitored, with the first obstetric ultrasound conducted at 24 weeks of gestation showing microcephaly, a single cerebral ventricular cavity, and laminated brain parenchyma with thalamic fusion, suggestive of alobar HPE (Figure 1). The parents were non-consanguineous, with no family history of malformation, tobacco use, drug exposure, or infections during pregnancy.

**Figure 2:**
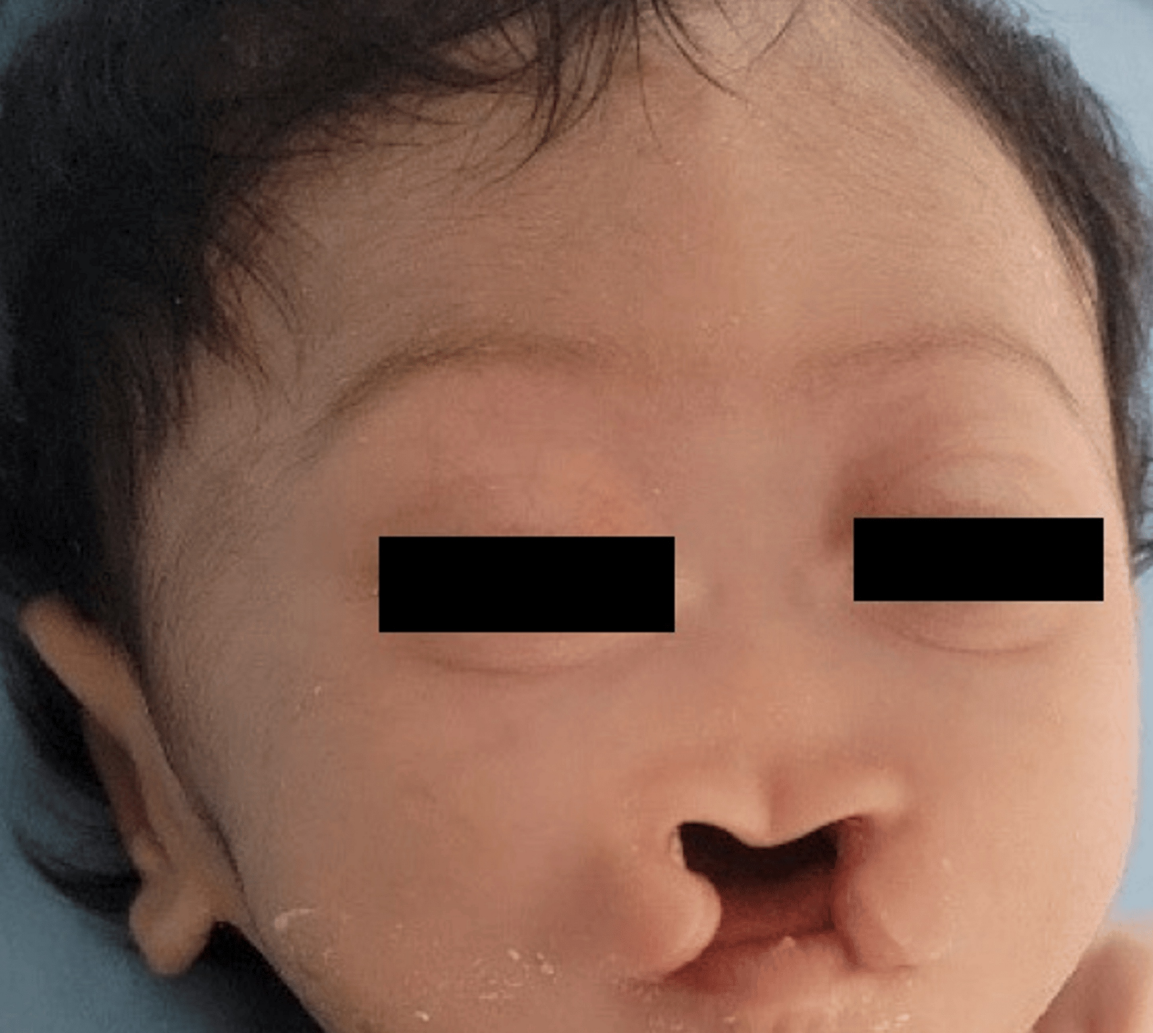
Newborn's face showing microcephaly, hypertelorism, hypoplasia of the nasal pyramid, premaxillary agenesis, median cleft lip-palate, and low-set ears

At clinical examination, the Apgar score was 7/10 at 1’ and 9/10 at 5’. Craniofacial anomalies observed included microcephaly, hypertelorism, hypoplasia of the nasal pyramid, premaxillary agenesis, a median cleft lip-palate, and low-set ears. The newborn also showed generalized hypotonia, difficulty sucking, and absent primitive reflexes. Cardiovascular and respiratory systems were clinically normal (Figure 2).

**Figure 3:**
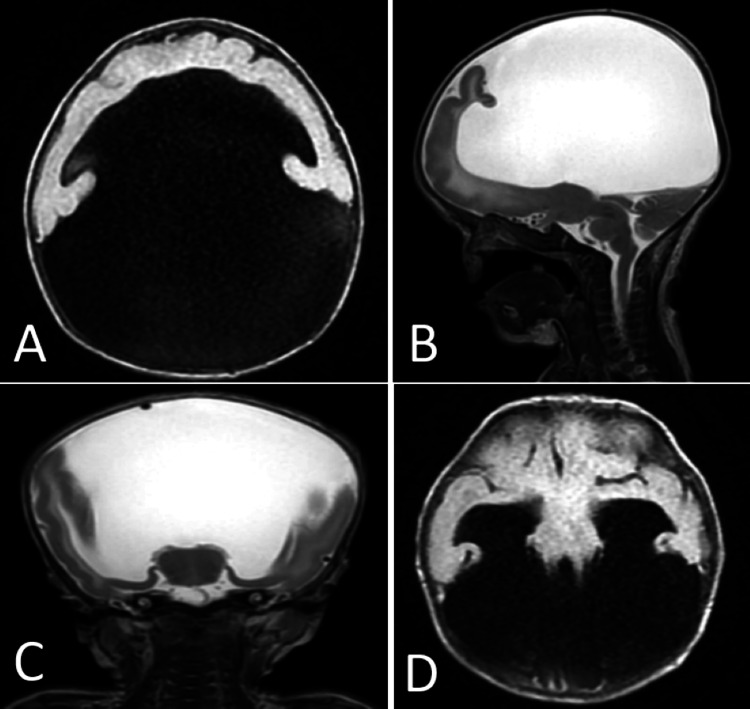
Brain MRI in axial T1 (A) and sagittal T2 (B) views shows residual brain parenchyma limited to an anterior-inferior lamina associated with a single ventricle communicating with a large dorsal cyst. Coronal T2 (C) and axial T1 (D) images show the absence of an interhemispheric fissure and non-separated central gray nuclei and thalami at the midline

The abdominal CT scan revealed no abdominal malformations, while echocardiography suggested asymmetrical hypertrophic cardiomyopathy without obstruction. Brain MRI showed significant ventricular system dilation, with a single ventricle communicating with a large dorsal cyst. The brain parenchyma was limited to a thin anterior-inferior lamina in the frontal and temporal lobes, with the absence of an interhemispheric fissure, corpus callosum, and olfactory bulbs. Additionally, the thalami were undifferentiated (Figure 3).

Genetic investigations were not conducted due to the parent's socioeconomic status. The newborn received necessary care in neonatology and was discharged with a guarded prognosis after the parents were informed. They were also advised on the importance of genetic counseling for future pregnancies.

## Discussion

HPE is a complex congenital malformation of the prosencephalon, characterized by structural brain abnormalities arising from midline differentiation and cleavage defects. It encompasses a broad spectrum of intracranial and craniofacial malformations affecting the midline [1]. HPE is the most common prosencephalic malformation, with a prevalence below 1 in 10,000 for live births, including stillbirths, and higher rates when pregnancy terminations are included [2]. Female predominance among live births is assumed to be due to the higher lethality of HPE in males [7].

During primary neurulation, the neural tube closes, forming three primary brain vesicles: the prosencephalon, mesencephalon, and rhombencephalon. In the subsequent ventral induction phase, occurring between the fifth and tenth weeks of gestation, these vesicles expand and divide into secondary vesicles, with the prosencephalon differentiating into the telencephalon and diencephalon. Disruptions in key signaling pathways, such as Sonic hedgehog (Shh), can lead to HPE [5]. Based on the degree of cerebral hemisphere differentiation, HPE is divided into two categories. The first, described by DeMyer and Zeman, includes the classical forms, ordered by severity: alobar, semilobar, and lobar [3]. The second category corresponds to the median interhemispheric variant, also known as syntelencephaly [4].

HPE is a multifactorial disorder characterized by significant etiological heterogeneity, influenced by environmental, genetic, teratogenic, and syndromic factors [6]. Chromosomal abnormalities are frequently associated with HPE, accounting for approximately 25% to 50% of cases, with trisomy 13 (Patau syndrome) being the most common. Additionally, between 18% and 25% of HPE cases occur within monogenic syndromes, including those with autosomal recessive inheritance. Genetic anomalies are predominant in its etiopathogenesis, with mutations identified in at least 14 genes, most commonly in HPE3 (Shh), HPE5 (Zic Family Member 2), SIX3, and TGIF [5]. Furthermore, environmental factors such as low socioeconomic status, maternal diabetes, alcohol consumption, retinoid use, and certain congenital infections, such as cytomegalovirus, also contribute to HPE risk [8].

Alobar HPE represents the most severe and common form, characterized by the absence of prosencephalic separation, resulting in a remnant of the primitive prosencephalon along the frontal midline without division into right and left lobes, and a primitive central monoventricle, sometimes associated with a prominent dorsal cyst. This condition is generally diagnosed via prenatal ultrasound or fetal MRI, but postnatal imaging is rare since most affected newborns are stillborn or have a short life expectancy [9]. MRI findings in alobar HPE reveal a complete absence of the interhemispheric fissure and undefined temporal lobes. Other midline structures, such as the superior sagittal sinus, cerebral falx, septum pellucidum, corpus callosum, third ventricle, anterior commissure, pituitary gland, and olfactory apparatus, are also absent. The optic nerves and chiasm may be absent, hypoplastic, fused, or normal, often with deficient myelination [5].

In our patient, the antenatal diagnosis of the malformation was made late, at 23 weeks of gestation, due to insufficient pregnancy monitoring. This delay contraindicated any therapeutic termination of the pregnancy. Although HPE was confirmed postnatally by a brain MRI performed at 32 days, an earlier diagnosis would have allowed for better management. However, even with early diagnosis, therapeutic termination of pregnancy in Morocco remains a sensitive subject, faced with ethical, moral, legal, and religious challenges.

The most severe facial malformations are associated with alobar HPE [1]. Clinically, surviving newborns present with severe neurological impairment and may experience feeding difficulties, neonatal seizures, infantile spasms, abnormal reflexes, autonomic nervous system dysfunction, diabetes insipidus, intellectual disability, and tone abnormalities with rigidity. Facial and cranial dysmorphism is almost always present, including microcephaly or macrocephaly, proboscis, cyclopia, hypotelorism, anophthalmia or microphthalmia, a flat or rudimentary nose, a single nostril, or a median cleft lip-palate, along with agenesis or hypoplasia of the premaxillary segment of the face [5].

The care of patients with HPE requires a multidisciplinary approach involving gastroenterologists, neurologists, neurosurgeons, and pediatric endocrinologists. Comprehensive evaluations of vision, hearing, feeding, and neurodevelopment are essential, as complications such as oromotor dysfunction, endocrine issues, hydrocephalus, and seizures are common and require monitoring. Movement disorders and spasticity, often seen in HPE, may benefit from therapies similar to those for cerebral palsy. Higher-functioning patients may also face neuropsychological challenges, for which psychological support or medication may be necessary [1].

The prognosis for HPE, particularly in its alobar form, is generally very poor. This form, the most severe, is often associated with a high mortality rate. Approximately 33% of affected newborns die within the first 24 hours, and 58% die within the first month. However, around 29% survive to the first year [10]. Survival is strongly influenced by the severity of cerebral and facial malformations, as well as the presence of other chromosomal anomalies or polymalformative syndromes. Children who survive beyond the neonatal period often have severe neurological impairments and serious medical complications [7].

## Conclusions

HPE is a complex congenital malformation presenting a wide range of clinical and radiological manifestations influenced by the severity of the anomaly. Early diagnosis, especially through fetal ultrasound, is crucial to optimize management and inform families regarding prognosis. Despite advances in imaging techniques, ethical and clinical challenges related to therapeutic termination remain important considerations in managing this pathology. Enhanced understanding of the etiopathogenic mechanisms of HPE and a multidisciplinary approach are essential to improving follow-up and support for affected children and their families.
